# The Young Innovators Program at the Eshelman Institute for Innovation: a case study examining the role of a professional pharmacy school in enhancing STEM pursuits among secondary school students

**DOI:** 10.1186/s40594-017-0081-4

**Published:** 2017-09-16

**Authors:** Adam D. Friedman, Carlos R. Melendez, Antonio A. Bush, Samuel K. Lai, Jacqueline E. McLaughlin

**Affiliations:** 1 Eshelman Institute for Innovation, UNC Eshelman School of Pharmacy, University of North Carolina at Chapel Hill, Chapel Hill, NC 27599 USA; 2 UNC Eshelman School of Pharmacy, University of North Carolina at Chapel Hill, Chapel Hill, NC 27599 USA; 3 UNC School of Education, University of North Carolina at Chapel Hill, 326 Beard Hall, Chapel Hill, NC 27599 USA

**Keywords:** STEM, Secondary school, High school, Research, Role model

## Abstract

**Background:**

Professional schools, such as schools of pharmacy, are rarely involved with promoting STEM interests among secondary school students. To address this shortcoming, the Young Innovators﻿ Program (YIP) was created to provide local secondary school students a summer immersive experiential program at the UNC Eshelman School of Pharmacy. The objective of this study was to assess the ability of the inaugural YIP to promote STEM interest, career awareness, and self-efficacy.

**Results:**

YIP interns maintained high levels of STEM interest, career awareness, and self-efficacy. In addition, they reported significant increases in their perceptions of having role models in science.

**Conclusions:**

Immersion in research laboratories and clinics at a school of pharmacy can promote high levels of STEM interest, career awareness, and self-efficacy and provide interns with STEM professional role models. Our findings support YIP's vision that professional pharmacy schools can play an influential role in recruiting secondary school students to STEM disciplines.

## Background

The United States (U.S.) pharmaceutical industry has experienced significant workforce shortages in the science, technology, engineering, and mathematics (STEM) workforce in recent years. In the biomedical science field alone, biomedical degree conferrals (bachelor’s, master’s, and doctorates) for 2015 numbered 109,896, whereas the number of new positions in professions that typically require these graduates, such as pharmaceutical manufacturing, scientific research, and hospitals, grew by 200,260 in 2016 (National Center for Education Statistics 2016, Table 325.20; Bureau of Labor Statistics 2015, Table NAICS 622100; Bureau of Labor Statistics 2016, Table NAICS 622100).

These workforce shortages are likely influenced by well-documented deficits in STEM education (President’s Council of Advisors on Science and Technology 2012; Radford et al. 2010; United States Census Bureau 2014). While the U.S. currently graduates about 300,000 bachelor and associate degrees in STEM annually (Radford et al. 2010), fewer than 40% of students intending to major in a STEM field persist in STEM until graduation (President's Council of Advisors on Science and Technology 2012). Further, only 29.3% of individuals with STEM bachelor’s degrees in the entire workforce were working in a STEM occupation in 2009 (President’s Council of Advisors on Science and Technology 2012) and 74% of recipients of STEM bachelor’s degrees in 2014 did not enter a STEM profession (United States Census Bureau 2014).

While shortages at the collegiate level are apparent, STEM deficits may be occurring as early as the 9th grade. In 2015, the U.S. ranked 30th in math and 19th in science out of the 35 member nations of the Organization for Economic Cooperation and Development (OECD) on the Programme for International Student Assessment (PISA) (DeSilver 2017). The U.S. PISA ranking was below the OECD average in math and near the OECD average in science, with both areas remaining relatively stable from prior years (DeSilver 2017). However, the U.S. was above the OECD average for the *share of students with science-related career expectations*, with 33% of boys and 43% of girls indicating that they expect to work in science-related occupations, compared to 25% of boys and 24% of girls on average from OECD countries. The U.S. also ranked higher than the OECD average for *motivation for learning science* (Gurria 2016)*.*

**Fig. 1:**
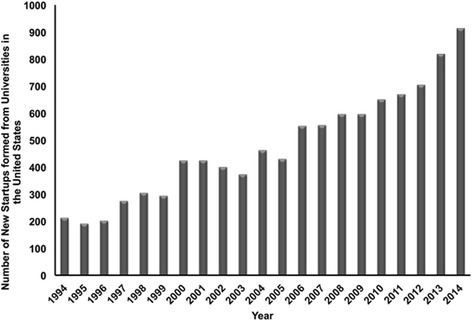
New startup companies formed from universities in the United States from 1994 to 2014

The benefits of immersive research experience for recruiting students into STEM degree programs and careers are well supported (Adedokun et al. 2013; Bell et al. 2003; Kenny et al. 1998; Millspaugh and Millenbah 2004; Nadelson et al. 2015; Pender et al. 2010). These experiences are especially critical for students at the secondary school level, who are beginning to explore career paths and engage in vocationally relevant activities. Indeed, in some cases, early laboratory experiences can help secondary students overcome various cultural, physical, and financial barriers impeding their transition to higher education and into STEM disciplines (Astin 1993; Baker and Taylor 1998; Blustein 2013; Downey and Ainsworth-Darnell 2002; Stolle-McAllister 2011; Strayhorn 2011; Tinto 1993; Tsui 2007; Wang and Eccles 2013). Universities and their associated health professions schools (e.g., medical schools, pharmacy schools) are often well positioned to offer immersive research experiences for students as highly concentrated centers of STEM activity, innovation, and entrepreneurship. As shown in Fig. 1, the growing number of spinout companies based upon STEM intellectual property developed in academic research laboratories and clinics is an observable result of this STEM activity (Association of University Technology Managers 2004, 2005, 2006, 2007, 2008, 2009, 2010, 2011, 2012, 2013, 2014).

Specifically, research programs in schools of pharmacy and pharmaceutical sciences often combine cutting edge research in chemistry and biology with innovative engineering and mathematics to develop next-generation medications (e.g., UNC Eshelman School of Pharmacy 2018; University of Michigan College of Pharmacy 2018; University of Minnesota College of Pharmacy 2018). Nevertheless, the role of pharmacy schools in the recruitment of secondary students into STEM remains largely unexamined. The purpose of this study is to describe the development, implementation, and evaluation of the Young Innovators﻿ Program at the University of North Carolina (UNC) Eshelman School of Pharmacy. This work was guided by the following research questions:

1. Does STEM interest and career awareness of secondary students change following participation in the YIP program?
2. Does STEM self-efficacy of secondary students change following participation in the YIP program?

### Methods

#### Research context: YIP program

The Eshelman Institute for Innovation, a part of the UNC Eshelman School of Pharmacy, launched the YIP program with the core belief that there is an underappreciated and largely unexplored role for professional schools to provide immersive STEM experiences to secondary school students. Through the program, we hoped to:

- Promote STEM interest, awareness, and self-efficacy by engaging students in a hands-on immersive experience based in a research laboratory
- Identify strategies that can be used to immerse secondary students in STEM-based research in the pharmaceutical sciences and pharmacy practice
- Demonstrate that schools of pharmacy can provide meaningful experiences to STEM secondary student recruitment during brief, experiential-based programming.

In the summer of 2016, the YIP Pilot immersed the first cohort (*n* = 17) of secondary students, termed interns, into research laboratories at the UNC Eshelman School of Pharmacy and provided a suite of activities aimed at exposing interns to a diversity of STEM careers and professionals (Table 1). Interns spent most of their time in laboratories across the school learning and developing experimental skills and research methods. Each participant was assigned a laboratory mentor (e.g., direct supervisor in the laboratory), preceptor (e.g., hosting professor), and clinical mentor (e.g., Doctor of Pharmacy student). Interns attended five expert panels on STEM pathways and careers titled Undergraduate Research, Graduate and Professional Students, Postdoctoral Researchers, Biotech Industry Leaders, and UNC Undergraduate Admissions.

**Table 1:** Weekly schedule for interns in the 2016 Young Innovators Program at the UNC Eshelman School of Pharmacy

Week^a^	Example activities
1	YIP Orientation Welcome Cookout
2	Undergraduate Education Panel ViiV Pharmacy Workshop 1
3	BeAM MakerSpace Lunch in downtown Chapel Hill, NC
4	Graduate/Professional Education Panel ViiV Pharmacy Workshop 2
5	Meeting with the Dean, UNC Eshelman School of Pharmacy
6	Postdoctoral Fellow Education Panel ViiV Pharmacy Workshop 3
7	UNC Undergraduate Admissions and tour of UNC campus Ice cream social
8	Central Inpatient Pharmacy and Cancer Hospital Infusion Pharmacy tour Lunch and learn with health-system pharmacist
9	FujiFilm Diosynth Biotechnologies Tour
10	Research Triangle Park Industry Leaders Panel YIP End-of-Summer Symposium

^a^Interns worked in laboratories each week and met regularly with YIP mentors and the program director

In addition, interns participated in the Innovation Challenge, a workshop that discussed the present and future of clinical pharmacy practice, as well as the concepts of problem-solving, failure, collaboration, and communication. As part of the Innovation Challenge, interns were asked to identify a limitation in the practice of pharmacy, discuss it with healthcare professionals, and develop possible solutions. Multiple tours allowed interns to observe STEM workplaces and careers: FujiFilm Diosynth Biotechnologies, Central Inpatient Pharmacy at UNC Hospitals, and the Chapel Hill Analytical and Nanofabrication Laboratory. At the 2016 End-of-Summer Research Symposium, each intern submitted a 2-page report in the style of an academic paper, gave a podium presentation of their individual laboratory research, and presented their solution to the Innovation Challenge project with their group.

#### Participants

YIP students were recruited from nine secondary schools located with 30 miles of the university. Teachers at these schools identified rising seniors that met YIP selection criteria, which included a minimum grade point average and interest in a summer internship. Intern candidates were asked for a one-page essay in which they described their interest in the program. Interns were also provided a list of participating YIP faculty mentors at this time and were tasked with identifying and justifying their first and second faculty mentor choices, from which program directors finalized intern, faculty assignments.

#### Data collection

All interns were asked to complete a pre-survey at the start of the program and a post-survey 2 months after the conclusion of the program. Survey participation was voluntary, consent was obtained, and no incentives were provided. The pre-survey and the post-survey incorporated previously validated instruments of STEM interest, self-efficacy, and career awareness (Kier et al. 2014; Milner et al. 2013; Tyler-Wood et al. 2010). The STEM Career Interest Test and STEM Career Self-Efficacy Test use 5-point Likert Scales (Milner et al. 2013; Armstrong et al. 2008; Day and Rounds 1997; Liao et al. 2008) while the STEM Semantics Survey uses 7-point scales to assess participant perceptions of the STEM disciplines (Tyler-Wood et al. 2010). The post-survey also included items about student experiences in the program and open-text questions about what students most enjoyed and found most challenging.

#### Data analysis

Descriptive statistics were computed for all items, and survey results are presented as (median, range). Wilcoxon Signed Rank test was used to examine differences between items on the pre- and post-survey. The significance level (*α*) was set at .05.

## Findings﻿

There were 17 YIP interns in 2016. Twelve participants identified as white (70.6%) while 5 participants identified Asian (29.4%). Nine of the participants are identified as being female (52.9%). Response rates for the pre-survey and post-survey were 100% (*n* = 17) and 94.1% (*n* = 16), respectively. On both the pre- and post-survey, participants indicated positive perceptions of STEM, with median responses on most items towards the top of the scale (e.g., *I am interested in careers that use science* (7, 2–7)). Responses increased significantly from the pre- to post-survey for the items *I have a role model in a science career* (5, 2–7 vs 7, 4–7, *p* = .016) and *I have a role model who uses technology in their career* (5, 2–7 vs 6, 4–7, *p* = .002)*.* On the post-survey, respondents indicated spending 4 to more than 12 h per week in an assigned laboratory and most commonly met with their laboratory mentor at least daily (*n* = 8), their preceptor at least once a week (*n* = 11), and their clinical mentor at least once a week (*n* = 12).

On a seven-point agreement scale, all respondents at least agreed (*n* = 16) that they *felt welcomed at the UNC Eshelman School of Pharmacy* (7, 6–7), *experienced an inclusive environment at this school* (7, 6–7), and *highly valued what I learned in this internship* (7, 5–7). In addition, most participants at least agreed that YIP experiences *positively influenced their desire to pursue a STEM or Information Communications Technology (ICT) career* (*n* = 13 at least agreed; 7, 4–7), *improved their knowledge in STEM or ICT* (*n* = 15 at least agreed; 7, 4–7), and *positively influenced their decision to pursue a higher education degree in STEM or ICT career* (*n* = 14 at least agreed; 7, 2–7).

In response to an open-text prompt about the most enjoyable part of YIP, one student responded that, “I enjoyed the hands-on, immersive component of the program the most. I can hardly describe the excitement I felt when I was told I would get to couple amino acids. Actually doing the coupling was even more exciting.” Another student stated, “I enjoyed that the YIP program introduced me into a field of study that I had no previous knowledge in. I also appreciated the opportunity to interact with many professionals within the university.” When asked about the most challenging aspects of YIP, one student commented, “…the most challenging was jumping into a highly technical, highly specific area of study that I knew nothing about. I had to learn quickly about very complicated concepts…” while another noted, “I found the questions my mentor asked me during my laboratory experience to be most difficult, though I wouldn’t change that if I could.”

## Discussion

In taking a first step towards demonstrating that schools of pharmacy can play a direct role in STEM secondary student recruitment, the YIP Pilot immersed interns in laboratories, clinics, and entrepreneurial spaces at the UNC Eshelman School of Pharmacy and engaged them in a wide range of activities aimed at exposing them to a diversity of STEM careers and professionals. Intern responses to the surveys suggest that interns frequently interacted with STEM professionals and gained role models as a result; engaged in real-world laboratory research, as evidenced by their time spent in laboratories; and enjoyed their experience at the school and in the YIP Pilot, as demonstrated by their valuation of the learning they received and sustained STEM interest 2 months after the program concluded.

It may be worth noting that interns participating in YIP entered the program with high levels of STEM interest, career awareness, and self-efficacy, which may have limited our ability to demonstrate a significant change in these items pre-survey to post-survey. The maintenance of STEM interest among the interns, however, is important because STEM active learning in classroom settings is fundamentally different from the YIP intern’s experience in research laboratories and clinics (Sadler et al. 2010). Though both classrooms and YIP can immerse the learner in active learning (Haak et al. 2011; Jensen et al. 2015; Slavin et al. 2001), the pursuit of real-world problems at a professional school oftentimes involves probing the unknown via hypothesis-driven research (Bell et al. 2003; Sadler et al. 2010). YIP challenged interns to pursue real, unsolved problems, and to apply their knowledge in the acquisition of novel findings via laboratory research, the treatment of human disease via clinical practice, and the translation of novel knowledge into usable products via healthcare entrepreneurship (Bell et al. 2003; Council, N. R. 1996). Therefore, it may be critical to maintain STEM interest among students as they transition from classroom case studies to research innovation––from students to problem solvers and future STEM professionals––in order to increase STEM recruitment (Seymour 2000).

It is possible that the maintenance of intern STEM interest may have been supported by their acquisition of role models. Based upon intern responses to questions regarding the frequency of their interactions with preceptors and mentors, it is assumed that intern preceptors and/or mentors became their role models. Role models who are STEM professionals are critical for the maintenance of student STEM interest and provide interns with the necessary encouragement to embark towards a STEM career (Erdogan and Stuessy 2015; Shin et al. 2016). If this assumption is correct, then these results would suggest that pharmacy schools can serve a direct role in the maintaining student STEM interest via the provision of role models during secondary student immersion in a multifaceted and innovative professional STEM environment. It is our hope that, in such a situation, these interns persist in their pursuit of a STEM career after transitioning to higher education: an outcome that could possibly reflect the direct role and effect that the UNC Eshelman School of Pharmacy had on STEM secondary student recruitment within the YIP Pilot intern cohort. More longitudinal data is required, however, to determine if such a result is correlated with, or influenced by, intern participation in the YIP Pilot.

To better understand the impact of the YIP program, data collection will continue during additional program iterations. Collecting and analyzing data from multiple intern cohorts may help overcome limitations associated with small sample sizes, which potentially resulted in underpowered statistical tests in this pilot. Ongoing relationships with YIP interns will enable us to track students over time to better understand the prolonged impact of the program. In addition, opportunity costs related to transportation and lost summer wages were an impediment to YIP for many students from underrepresented (URM) and low socio-economic status (SES) backgrounds. As such, expanded support (e.g., summer stipends, travel) will be provided to interns in future cohorts to mitigate opportunity costs associated with program participation and make the program more accessible for students from these groups.

Relatedly, several opportunities for programmatic improvement have been identified for future iterations of YIP. Since pre-college summer bridge programs based on attrition and persistence theories have increased URM student participation and persistence in STEM fields, YIP will modified to include additional recruitment strategies (e.g., coordinated trips to low SES schools to inform counselors and teachers about the program) and evidence-based programming to promote the engagement and persistence of URMs in STEM (Stolle-McAllister 2011; Strayhorn 2011; Tsui 2007). The participation of teachers from secondary education will also be expanded since these individuals play a critical role in the culture and instruction that fosters STEM learning and interest (Logan and Skamp 2008; Swarat et al. 2012; Wang and Eccles 2012). Teacher engagement will include many of the same summer activities attended by interns, with additional weekly workshops and seminars that provide training and curriculum development support for translating and implementing cross-cutting STEM methods into their own secondary school STEM courses. Subsequent research will examine perspectives from the secondary sector regarding the impact of YIP on STEM learning in secondary education.

## Conclusions

The YIP program provided immersive research experiences in academic laboratories within a school of pharmacy. The findings suggest that professional schools can play an important role in promoting STEM awareness, providing real-world STEM experience, and connecting secondary students with STEM mentors well before they enroll in college. Given the innovative and often entrepreneurial nature of research in higher education, it is our hope that the results of this pilot serve as a model for other schools positioned to create similar programming that supports STEM recruitment.

## Abbreviations

ICT: Information Communications Technology
STEM: Science, technology, engineering, and mathematics
